# *Notes from the Field:* Lead Poisoning in a Family of Five Resulting from Use of Traditional Glazed Ceramic Ware — New York City, 2017–2022

**DOI:** 10.15585/mmwr.mm7122a3

**Published:** 2022-06-03

**Authors:** Paromita Hore, Kolapo Alex-Oni, Nevila Bardhi, Slavenka Sedlar

**Affiliations:** 1 Bureau of Environmental Disease and Injury Prevention, New York City Department of Health and Mental Hygiene, New York, New York.

The New York City (NYC) Department of Health and Mental Hygiene (DOHMH) receives blood lead test results for NYC residents and conducts investigations of child and adult lead poisoning cases (*1*). Routine blood lead screening of a child in 2017 ultimately led to the discovery of a family of five with blood lead levels at or above the CDC blood lead reference value at that time of 5 *µ*g/dL (range = 5–53 *µ*g/dL) in November 2020.*^,†^ Case investigations revealed that the elevated blood lead levels were associated with the use of traditional, glazed ceramic ware. DOHMH intervention resulted in a decrease in blood lead levels for all family members (range = 1–6 *µ*g/dL at last measurement dates).

In September 2017, during routine screening by a health care provider, a child aged 3 years was found to have a blood lead level of 7 *µ*g/dL (Figure). At the time, DOHMH’s threshold for an in-home inspection was 10 *µ*g/dL; therefore, a home inspection was not conducted. DOHMH sent letters to the child’s guardians and to the medical provider recommending follow-up testing for the child, testing of family members, and providing guidance on how to reduce lead exposure, including avoiding use of clay pots and dishes from other countries. In 2018, the child received a blood lead test result of 5 *µ*g/dL. Letters were sent to the family and to the medical provider. A DOHMH home inspection was offered, but the family declined.

**FIGURE:**
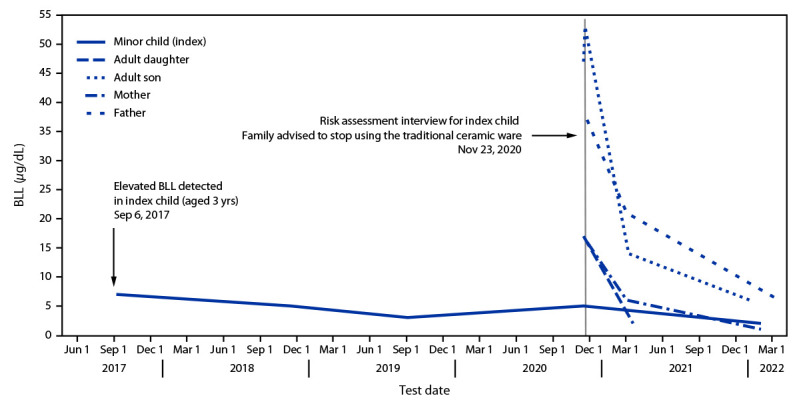
Blood lead levels in members of a single family with exposure to traditional glazed ceramic ware — New York City, 2017–2022 **Abbreviation:** BLL = blood lead level.

In November 2020, the child’s blood was retested for lead, and, as encouraged by the family physician and DOHMH, blood samples from the child’s two adult siblings were also tested; all three had blood lead levels at or above 5 *µ*g/dL (5, 17, and 53 *µ*g/dL, respectively). Shortly thereafter, the mother and father received elevated blood lead test results (16 and 37 *µ*g/dL, respectively).

During follow-up risk assessment interviews*,* DOHMH learned that the family was using traditional ceramic ware purchased in Mexico for cooking, storing meals, and making coffee. DOHMH screened the ceramic ware using an X-ray fluorescence device (Viken Detection). The glazed interior measured 15.7 mg of lead per cm^2^, a level with the potential to leach substantial amounts of lead, particularly when used for cooking (*2*). The family again declined a home inspection; consequently, DOHMH was unable to ascertain potential exposures to other lead sources, including lead paint, for the index child. Occupational sources were excluded for the adults. The mother reported that she sometimes used Mexican spices for cooking, and the father reported being engaged in household renovation activities. The family did not provide spice samples, and because they did not agree to a home inspection, it is not known whether or to what extent these potential sources might have contributed to the poisonings. The family stopped using the traditional, glazed ceramic ware for food and drinks after speaking with DOHMH investigators, and their blood lead levels declined to 2–21 *µ*g/dL within 3–4 months and to 1–6 *µ*g/dL after 14–16 months.

Lead can cause serious health effects in both children and adults; therefore, exposure to known lead sources should be avoided. Traditional ceramic ware from around the world has been found to contain lead at levels thousands of times higher than regulatory limits in the United States (*3*). The lead used for aesthetic and other purposes on the ceramic ware’s glaze or paint can transfer to foods or drinks that are prepared, served, or stored in these products, placing users at risk for lead exposure. DOHMH has investigated lead poisoning in children and adults associated with ceramic ware purchased in Ecuador, Mexico, Morocco, Turkey, the United States, and Uzbekistan (*3*). Continued efforts to raise awareness about lead hazards associated with traditional ceramic ware are needed among potential users and health care providers. The family in this report was unaware of the potential for ceramic ware to contain lead, despite previous DOHMH guidance. Although DOHMH has taken enforcement actions to stop NYC businesses from selling lead-containing ceramic ware, this does not eliminate the hazard because families often bring such items from their home countries, as was the case for the family described in this report. In September 2021, DOHMH issued a press release (*3*) and health advisory (*4*) concerning the risk for lead exposure from traditional ceramic ware. A similar press release had been issued in May 2017 (*5*). Ultimately, source control (i.e., eliminating use of lead in ceramic glazes) is needed, which requires the engagement of global stakeholders. This investigation highlights the importance of testing blood lead levels of all household members when one member receives a diagnosis of an elevated blood lead level. In addition, local health departments should conduct a holistic risk assessment that examines multiple potential sources of lead exposure.

## Footnotes

*
https://www.cdc.gov/nceh/lead/default.htm

## References

[1] Hore P, Ahmed M, Nagin D, Clark N. Intervention model for contaminated consumer products: a multifaceted tool for protecting public health. Am J Public Health 2014;104:1377–83. 10.2105/AJPH.2014.301912 24922141 PMC4103255

[2] Food and Drug Administration. Regulatory guidance for lead in ceramic ware. Silver Spring, MD: US Department of Health and Human Services, Food and Drug Administration; 2005. https://www.fda.gov/media/71764/download

[3] New York City Department of Health and Mental Hygiene. Health department issues lead warning to New Yorkers: avoid using traditional ceramic ware for food and drinks; may contain lead. New York, NY: New York City Department of Health and Mental Hygiene; 2021. https://www1.nyc.gov/site/doh/about/press/pr2021/dohmh-warning-for-lead-ceramic-ware.page

[4] New York City Department of Health and Mental Hygiene. 2021 Health Advisory #: 37 elevated levels of lead in traditional ceramic ware. New York, NY: New York City Department of Health and Mental Hygiene; 2021. https://www1.nyc.gov/assets/doh/downloads/pdf/han/advisory/2021/lead-ceramic-ware.pdf

[5] New York City Department of Health and Mental Hygiene. Health department warns New Yorkers about clay pottery with extremely high levels of lead [Press release]. New York, NY: New York City Department of Health and Mental Hygiene; 2017. https://www1.nyc.gov/site/doh/about/press/misc/pr035-17.page

